# Simulation of Rapid Thermal Cycle for Ultra-Fast PCR

**DOI:** 10.3390/s22249990

**Published:** 2022-12-18

**Authors:** Zhuo Yang, Jiali Zhang, Xin Tong, Wenbing Li, Lijuan Liang, Bo Liu, Chang Chen

**Affiliations:** 1 School of Microelectronics, Shanghai University, Shanghai 201800, China; 2 Shanghai Industrial μTechnology Research Institute, Shanghai 201800, China; 3 State Key Laboratory of Transducer Technology, Shanghai Institute of Microsystem and Information Technology, Chinese Academy of Sciences, Shanghai 200050, China; 4 Shanghai Academy of Experimental Medicine, Shanghai 200052, China

**Keywords:** PCR system, Finite Element Methods, microfluidic chip, heat resistor

## Abstract

The polymerase chain reaction (PCR) technology is a mainstream detection method used in medical diagnoses, environmental monitoring, food hygiene, and safety. However, the systematic analysis of a compact structure with fast temperature changes for an ultra-fast PCR device that is convenient for on-site detection still lacks investigation. To overcome the problems of low heating efficiency and non-portability of PCR devices currently used, a miniaturized PCR system based on a microfluidic chip, i.e., lab-on-chip technology, has been proposed. The main objective of this paper is to explore the feasibility of using a heat resistor that can reach a fast heating rate and temperature uniformity combined with air cooling technology for rapid cooling and to investigate the influences of various pattern designs and thicknesses of the resistor on heating rates and temperature uniformity. Additionally, a PCR chip made of various materials with different thermal properties, such as surface emissivity, thermal conductivity, mass density, and heat capacity at constant pressure is analyzed. In addition to the heat loss caused by the natural convection of air, the radiation loss of the simulation object is also considered, which makes the model much closer to the practical situation. Our research results provide a considerable reference for the design of the heating and cooling modules used in the ultra-fast PCR protocol, which has great potential in In Vitro Diagnosis (IVD) and the PCR detection of foodborne pathogens and bacteria.

## 1. Introduction

The PCR technology, invented by Mullis in 1985, refers to a process combined with the denaturation, annealing, and extension of DNA fragments at specific temperature profiles for the detection of target DNA [1]. Various PCR protocols (as advanced technologies) are available on the market due to their wide applications in the fields of medical diagnosis [2,3], environmental monitoring [4,5], and food hygiene and safety [6,7,8]. The current micro-PCR chip introduced modern micro (nano)-fabrication to the PCR system, which enabled fast detection on a microfluidic chip with little reagent consumption. It leads to portable and hand-held devices for convenience in urgent applications.

At present, micro PCR chips are mainly divided into two types: static microcavity PCR chips [9], which rely on the rapid temperature response of a heating module to achieve nucleic acid amplification; and continuous flow PCR (CF-PCR) chips [10], which drive the reagent to circulate in the microfluidic chip above the heating module with various temperature regions to implement nucleic acid amplification. The nucleic acid amplification in the static microcavity PCR chip originates from the temperature rising and falling of the target DNA fragments. The accurate temperature control of the PCR chip is critical for the quantitative analysis of nucleic acid amplification. Up to date, many researches on the design and fabrication methods of heating modules are available in publications and real products, including micro-resistors [11], Peltier devices [12], laser heating [13], and electromagnetic wave heating [14]. Yin et al. [15] fabricated a thermal cycle platform on a PCB, with a Peltier device with a maximum heating rate of 30 °C/s and a maximum cooling rate of 10 °C/s, which successfully used the platform to quickly distinguish positive and negative cases in clinical samples and rapidly quantify the viral load in each sample. Neuzil et al. [16] designed a silicon-based micro-cantilever droplet PCR chip to amplify the full-length cDNA template obtained by reverse transcription (RT) of RNA using random hexamers, which used thin film heating and natural cooling to make the temperature rising and falling rates exceed 100 °C/s. Shaw et al. [17] developed a microwave-mediated non-contact DNA amplification thermal circulator with a heating rate of 65 °C/s and average power consumption of 500 mW, in which the PCR was successfully performed for the amplification of the Amelogenin locus. Chen et al. [14] showed a centrifugal microfluidic platform based on a wireless induction heater. A temperature of 93 °C was achieved in the heater when resonated with 0.49 W of radiofrequency field (RF) output power. Selective sterilization of *Escherichia coli* through wireless heating was also used in the PCR system for thermal cycling. Burger et al. [13] presented an infrared (IR) thermocycler with closed-loop temperature control to perform fast PCR in centrifugal microfluidics. IR radiation enabled the direct heating of aqueous reagents in cavities of microfluidic polymer film disks with high-temperature gradients. The average heating rate reached 4 °C/s and the average cooling rate reached 1.3 °C/s. The heating system of the continuous flow PCR chip is composed of two or three heating modules with different temperatures. Yang et al. [18] used two aluminum heating blocks controlled by two independent proportional–integral–differential (PID) controllers with a 0.1 °C temperature accuracy to make the heating system detect periodontal pathogens. Moreover, they built a microfluidic system based on a CF-PCR array microfluidic chip and successfully realized simultaneous amplification of P.g, T.d, and T.f in this microfluidic chip within 8 min and 5 s. Talebi et al. [19] used the easily-processed PCB resistor heating sheet to stick to a PCR chip. By properly designing meander-shaped electrodes, relatively good temperature uniformity and high PCR efficiency were realized. The fabricated microchip was tested via 88 base-pair DNA samples from the human housekeeping gene with a power consumption of less than 0.82 W; a relatively remarkable amplification efficiency was achieved. Jiang et al. [11] employed two PMMA boards to clamp the CF-PCR chip with two resistance heating films to form a heating system that enabled the miniaturization and integration of the whole system at a reduced volume. They developed a microfluidic device that combined high-throughput continuous-flow PCR and DNA hybridization for the detection of various bacterial pathogens.

In previous studies, two heating methods were studied extensively, including contact heating and non-contact heating involved a more direct way of transferring heat and fewer losses than non-contact heating, so it is more established for practical applications. In previous studies on contact heating, either the large size made it difficult to carry or the heating system could not be controlled by humans throughout. In the study related to microheaters, although the heating system was smaller, a systematic study for the heating module and the selection of materials for the chip were lacking. Here, we propose a systematic simulation study about this.

In this paper, a systematic simulation of a static microcavity chip based on specific materials with excellent thermal conductivity and biocompatibility properties is proposed. The integrated chip consists of a transparent glass cover at the top surface for sealing the fluidic biology reagents in purpose-designed microchannels in a microcavity on a microfluidic chip, and a patterned resistance microheater attached to the bottom of the microfluidic chip to provide a heat source for the amplification of nucleic acids of the DNA fragments. The special design of the microheater pattern enabled ultra-rapid rising of the temperature for DNA reagents, and together with forced air cooling around the integrated chip sealed in a small volume, very rapid heating and cooling rates and good temperature uniformity of the micro-fluidic chip could be achieved in the simulation. The finite element method (FEM) was used to solve the Fourier heat transfer equation for the integrated microfluidic chip and patterned resistance heater in this work, and it disclosed the space temperature distribution of the simulation objects under an electrically driven heat source of the microheaters with various patterns. For an accurate simulation of the system under both static conditions and transient conditions, the heat loss not only obtained the forced convection of cooling air involved but also considered the radiation emission loss of high-temperature objects combined with different materials based on Stefan–Boltzmann’s law [20].

## 2. Design and Method

**Figure 1:**
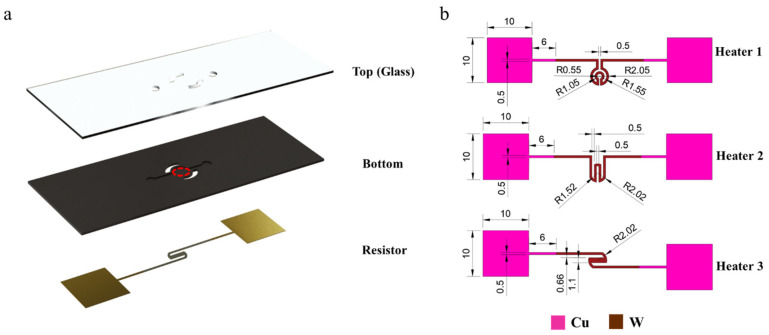
(**a**) The structure of the chip and the surface inside the red circle are the measurement areas. (**b**) Schematic diagram and specific sizes of three resistor patterns (mm).

As presented in Figure 1a, the integrated PCR chip was combined with a top glass cover and a bottom microfluidic chip, with microchannels and microcavities on one side and a specially designed resistor pattern on the other side. We chose this geometry and material combination for the simulation studies because they can be easily translated into a functional device experimentally using semiconductor processes. The microchannels and microcavity for containing biological reagents could be fabricated by plasma etching of a specific substrate made by Si, AlN, SiC, SiO_2_, or Al_2_O_3_, and the patterned micron heat resistor together with circuits could be fabricated by a thin metal film deposition (here tungsten (W) was selected as the heater material and copper (Cu) was selected as the electric connection. However, for Si and SiC substrates, an insulation layer as SiO_2_ film should be deposited on the back side at first), with photolithography and metal etching sequentially on the other side. The front side of the microfluidic chip and the pattern of the heating resistor are shown in Figure 1a,b. The geometrical parameters of the simulation object are displayed in Table 1. For a comparison study to find an optimized heating solution, heat resistors with three different patterns were designed as the Joule heat source for the heat transfer calculation (Figure 1b). The same voltage was supplied to the three heaters with various patterns in the simulation.

For the calculation of the temperature distribution, both at the transient condition and static condition of this simulation object, the Fourier heat transfer equation together with the FEM method was implemented. The general form of the governing equation for Fourier heat transfer is displayed below, with an assumption that the thermal diffusion coefficients of the materials involved were isotropic [20],

(1) $$\rho c\frac{\partial T}{\partial \tau }=\frac{\partial }{\partial x}\left(\lambda \frac{\partial T}{\partial x}\right)+\frac{\partial }{\partial y}\left(\lambda \frac{\partial T}{\partial y}\right)+\frac{\partial }{\partial z}\left(\lambda \frac{\partial T}{\partial z}\right)+\dot{\Phi }$$

where *T* is the temporal temperature depending on space and time (K), *ρ* is the mass density (kg/m^3^), *c* is the specific heat (J/(kg·K)), $\dot{\Phi }$ is the internal heat source (W/m^3^), and *λ* is the thermal conductivity (W/m^3^).

(2) $$\frac{\partial Q}{\partial t}=I^{2}\cdot R$$

where *t* is the time (s), *Q* is the heat source dependent on space (J), *I* is the electric current (A), and *R* is the resistance (Ω).

Considering the thermal losses from the heat radiation, natural convection, and the convection of forced air cooling, the other three terms should be added to the Fourier heat transfer equation,

(3) $$\Phi =\varepsilon A\sigma T^{4}$$

where *Φ* is the heat transfer rate (W), *T* is the black body’s thermodynamic temperature (K), *σ* is the Stefan–Boltzmann constant (5.67 × 10^−8^ W/(m^2^·K^4^)), *A* is the radiant surface area (m^2^), and *ε* is the surface emissivity.

(4) Φ=hAΔT

where *Φ* is the heat transfer rate (W), Δ*T* is the temperature difference (K), *A* is the surface area (m^2^), and *h* is the convective heat transfer coefficient (W/(m^2^·K)).

(5) $$\rho C_{p}\left(\frac{\partial T}{\partial \tau }+u\frac{\partial T}{\partial x}+v\frac{\partial T}{\partial y}+w\frac{\partial T}{\partial z}\right)=\lambda \left(\frac{\partial^{2}T}{\partial x^{2}}+\frac{\partial^{2}T}{\partial y^{2}}+\frac{\partial^{2}T}{\partial z^{2}}\right)$$

where *T* is the temporal temperature depending on time (K), *ρ* is the mass density (kg/m^3^), *C_p_* is the specific heat (J/(kg·K)), and *λ* is the thermal conductivity (W/m^3^).

The surface of the whole chip exposed to the air was added with the boundary condition of the natural air convection. The surface heat transfer coefficient of the natural air convection was roughly in the range of 1~10 w/(m^2^·k), which was taken as 5 w/(m^2^·k) in the simulation. At the same time, the outer surface of the chip was also added the boundary conditions of external radiations to move close to the the practical situation in the thermal simulation.

The materials considered as the substrates for the fabrication of the microfluidic chip and depositing of the resistance heater were displayed in Table 2, to satisfy the demands of the governing equation. Here, five different materials, such as Si, SiC, AlN, Al_2_O_3_, and SiO_2_ were considered as the materials for the substrate, for the reason that those were commonly used materials in academics, additionally there existed a huge contrast contrast of the thermal properties existed among them [21]. The main difference in the thermal properties for these five materials as substrate was the thermal conductivity and the surface emissivity, which was supposed to result in a contrast between the microfluidic chips made by each material. As the convection resulting from heat transfer was considered in the simulation, a cubic region containing air with the size of 80 mm × 80 mm × 50 mm was introduced to the geometrical model. The flow rate of the air was set at 5.390625 m/s on the Z-axis and 8.625 m/s on the X-axis, which was generated with a common fan.

## 3. Results and Discussion

**Figure 2:**
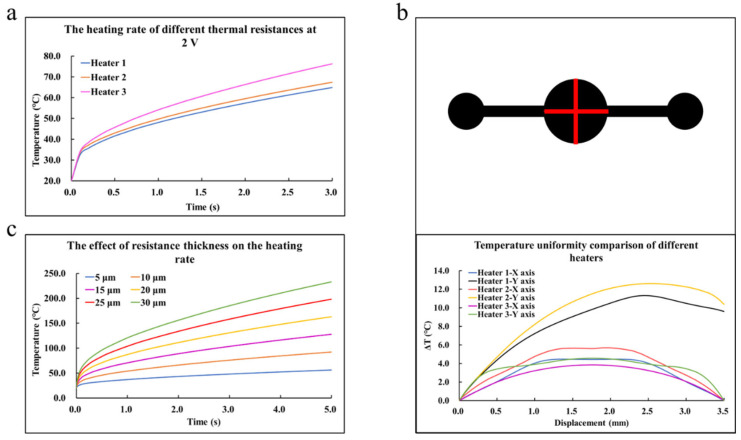
(**a**) The heating rates of different heat resistors at 2 volts. (**b**) X-axis and Y-axis lines were made on the surface of the microcavity, and the temperature gradients on the lines were measured to express the temperature uniformity of the microcavity surface with the temperature difference. (**c**) The influence of the thickness of resistors on the heating rate.

**Figure 3:**
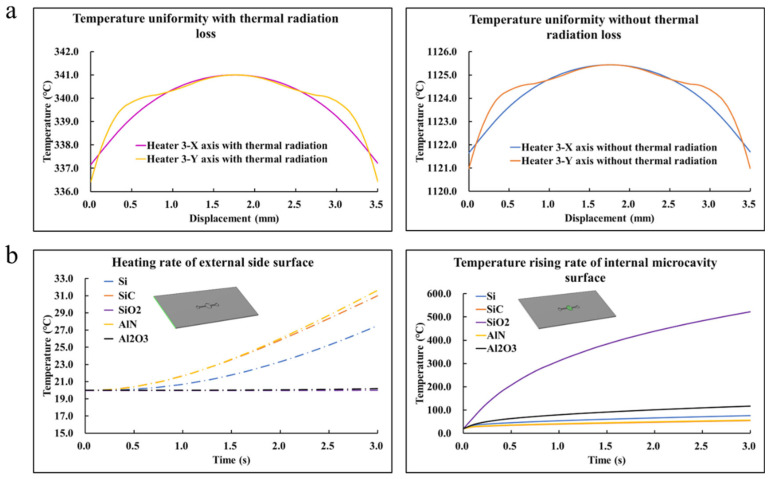
(**a**) Comparison of the effect of thermal radiation losses on simulation results. (**b**) Relationship between heating rates of the microcavity surface/external side surface and thermal conductivity of materials.

**Figure 4:**
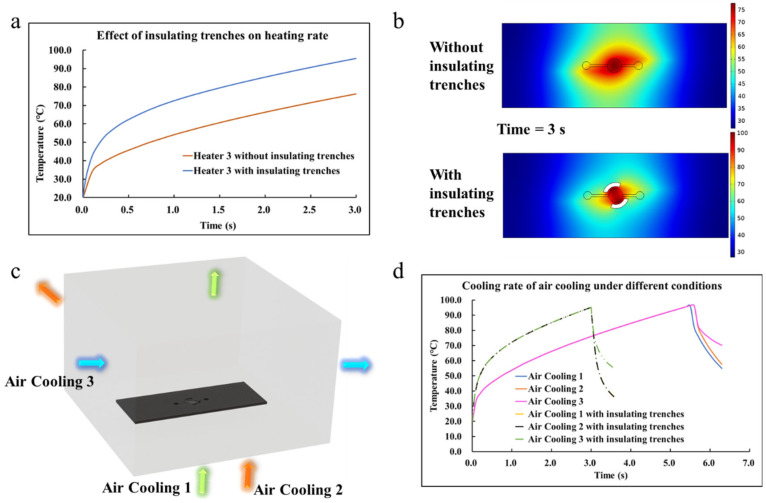
(**a**) The influence of insulating trenches on the heating rate. (**b**) The contrast of simulation results in temperature distribution. (**c**) There are three air cooling plans: air cooling 1—the green one chooses the lower side as the inlet and flows out from the upper side, air cooling 2—the orange one employs the lower side as the inlet and flows out from the horizontal side, and air cooling 3—the blue one uses the horizontal side as the inlet and flows out from the other side. (**d**) The cooling rates of air cooling under different conditions.

The simulation results are presented in Figure 2, Figure 3 and Figure 4. The heat rate at the transient condition and the temperature distribution over the microfluidic chip at the static condition for various patterns of the heating resistor are displayed in Figure 2; Additionally, the optimized heating solution for the same microfluidic chip was figured out. The key simulation results of the three heaters are shown in Table 3. The influences of the microfluidic chips made by different materials, such as SiO_2_, Al_2_O_3_, Si, SiC, and AlN with a huge contrast in thermal properties, are shown in Figure 3, and the effect of thermal radiation in the simulation was also carried out. To investigate how the hollowed insulating trenches around the cavity of the microfluidic chip affected the heat transfer at transient conditions, a comparative study of the heat rate for the structure with/without the insulating trenches is described in Figure 4. Moreover, the key simulation results of the three cooling methods are shown in Table 4.

In the simulation studies shown in Figure 2, the bottom material of the chip was Si, and the thickness of the microheater defaulted to 10 μm. As presented in Figure 2a, it was obvious that the average temperature in the empty cavity of the microfluidic chip followed the same rule while increasing with the times of all three design patterns of the heating resistor. At the beginning of the heating process within 0.1 s, the temperature appeared linear to the time, which indicated an almost constant heating rate during this period. In case the time was over 0.1 s, the nonlinear phenomena came out in the variation trend of the transient temperature and the heating rate values slid down a lot to deviate from the initial slopes. It was evident that Heater 3 showed an advantage over the other two designs to keep a higher heating rate after the inflection point at 0.1 s. The heating rate of Heater 1 was 14.95 °C/s, while the heating rate of Heater 3 was 18.77 °C/s. Furthermore, the heating rate differences between the three heaters showed similar trends with different materials. The simulations using different materials were carried out under the same environmental conditions, and the heating rate differences between the three heaters with Si mainly depended on the structural changes between the heaters. As shown in Figure 2b, the temperature difference was used to describe the temperature uniformity of the three patterns. It was clear that the temperature gradients of the cavity surface on the Y axis heated by Heaters 1 and 2 were higher and unsymmetrical due to the geometric construction of Heaters 1 and 2. The temperature gradients on the X-axis heated by all resistors were symmetrical. Among them, the temperature uniformity of Heater 3 was the best. The maximum temperature differences of Heater 1, Heater 2, and Heater 3 were 11.32 °C, 12.61 °C, and 4.58 °C, respectively. To summarize, Heater 3 was the most suitable heating part due to its advantages in the heating rate and temperature uniformity. Figure 2c shows the simulation results in different thicknesses of Heater 3. It could be seen that the heating rate of the empty cavity surface of the microfluidic chip was proportional to the thickness of the resistor. The heating rates of the resistors with thicknesses of 5 and 30 μm were 7.23 °C/s and 42.65 °C/s, respectively. Considering the process cost comprehensively, the resistor thickness was selected as 10 μm while the larger voltage applied could also have a faster heating rate. Based on the analysis of Figure 2a,b, the designed geometric structure of the resistor should be as symmetrical as possible.

Baesd on previous work, it was determined that the design of Heater 3 was used for the chip heating part, and the resistor thickness was set as 10 μm. Figure 3a illustrates the temperature distribution of the surface of the empty cavity on the X-axis and Y-axis with/without the thermal radiation and the same rule of both temperature uniformities at static conditions was presented. A huge contrast could be seen in Figure 3a, where the maximum temperature on the surface of the empty cavity reached about 341 °C with the thermal radiation at static conditions while the one without thermal radiation attained about 1125 °C. The obvious difference described the importance of considering the surface emissivity of materials during the thermal simulation. The bottom material of the chip was scanned with Si, SiC, AlN, Al_2_O_3_, and SiO_2_ due to the enormous contrast between the thermal conductivity and their excellent biocompatibility. In previous related simulations [26,27], researchers typically considered the heat loss caused by the natural convection of air, while the radiation loss also needed to be taken seriously, which was proved in Figure 3a. As can be seen in Figure 3b, it was found that the heating rate of the microcavity surface did not improve with the increased thermal conductivity as expected, which was inversely to it. In this regard, Equations (6) and (7) are used to show that the materials are related to the thickness, cross-sectional area, and thermal conductivity:

(6) r=d/(λs)

where *r* is the heating resistor of the material (Ω), *d* is the thickness through which heat passes (m), *λ* is the thermal conductivity of the material (W/(K*m)), and *s* is the surface area in which heat passes (m).

(7) r=(T2−T1)/P

where *T*2 is the temperature at one end, *T*1 is the temperature at the other end, and *P* is the heat source power.

Since the resistor was set as the bottom heat source, which was *T*1 by default, the rising of the heating resistor increased the temperature difference between the two sides of the bottom material of the PCR chip. In short, while the thermal conductivity of the bottom material decreased, the surface temperature of the microcavity increased. Of course, this is because the microcavity was in a closed state, which was sealed by a glass sheet; there was only a little loss on the surface of the microcavity. The outer surface of the chip side was exposed to the environment, which has a large heat loss. Therefore, the heating rates of the outer surface of the chip side were obtained by combining the surface emissivity and the thermal conductivity of the materials.

Cai et al. [28] designed the insulating trenches around the microcavity of the chip to achieve rapid temperature rising and falling, and the amplification could be completed in 5 min. In the simulation studies in Figure 4, Si was selected as the bottom material of the chip due to its good thermal properties and biocompatibility, and the resistor thickness was set as 10 μm. As figured out in Figure 4a, it could be seen that the heating rates follow the same rule as time goes by. The inflection point of the condition without insulating trenches was more obvious than the one with insulating trenches. It was evident that the heating rate with insulating trenches was significantly faster than the one without insulating trenches. As shown in Figure 4b, it was also found that the design of insulating trenches with the appropriate shape and size on the chip could make the local temperature distribution of the microcavity surface in the center of the chip visibly concentrated. While the air with high thermal resistance replaced the previous solid part, heat would have been more difficult to diffuse outward. The heating rate of Heater 3 with insulating trenches was 25.2 °C/s, and it was 6.43 °C/s faster than the one without insulating trenches. Moreover, the addition of insulating trenches can change the local temperature with different materials. However, it should be noted that the use of other materials requires minor changes to the shape and size of the tank depending on the specific situation.

There were two steps of transient calculations when analyzing the cooling of the chip. The first step was to carry out the heating simulation mentioned before, stop heating at about 95 °C, and then take its final result as the initial value of the second step to drive the simulation of forced air cooling. As proposed in Figure 4c, an air domain was set around the chip during the cooling simulation, and the distance between the bottom of the air domain and the bottom of the chip was 5 mm. Since the light sources and cameras of qPCR-chip products, which are generally detected through fluorescence, are normally installed at the top, the air inlet on the upper side was not considered.

The flow rate was 5.390625 m/s when the lower part was used as the inlet, and the flow rate was 8.625 m/s when the side was used as the inlet. In this simulation, a total of three air-cooling flow plans were considered. As supported in Figure 4d, it was obvious that the heating rates of the conditions with/without insulating trenches followed the same rule while increasing with time. At the beginning of the cooling process, the temperature appeared linear to the time evolution, which described an almost constant cooling rate during this period. Then the nonlinear phenomena appeared in the variation trend of the transient temperature and the cooling rate values slid down a lot to deviate from the initial slopes. Among them, the downtrend of air cooling 3 with/without insulating trenches was the most obvious. It was found that the cooling rate of air cooling 1 was the fastest, which was 51.8 °C/s, and the cooling rate of air cooling 2 was 48.44 °C/s. Since the insulating trenches were set so that the air could flow around the microcavity, the cooling rates of air cooling 1 and air cooling 2 became almost the same, reaching 98.76 °C/s and 99.24 °C/s, respectively. The benefits of setting insulating trenches to heat dissipation are proven.

## 4. Conclusions

This work was based on the systematic simulation of the selection of materials for microfluidic chips and heating and cooling modules. Three different heating patterns for application in a PCR protocol were designed and studied by the FEM method, with consideration of air convection. The comparative study presented the third design with a simple S-shaped heat resistor showing a higher heating rate under the same load voltage, for heating the disk-like microcavity in the microfluidic chip. The study on the thickness of the S-shaped heat resistor uncovered that a linear relationship between the heating rate and thickness values of the designed resistor existed. The addition of the insulating trenches around the cavity benefitted the improvement of the heating rate by limiting the heat loss by conduction. This also made the distribution of local temperature more concentrated. However, this conclusion is obtained in the simulation of a silicon-based chip, and the shape and size of the trenches may need to be slightly modified according to the specific situation when using other materials. Considering air cooling by convection, it was found that the thermal solution with specially designed insulation trenches showed a much faster cooling rate in the temperature profile. In addition, a vertical flow direction of the forced air from the bottom of the microfluidic chip to its top suggested a much higher cooling rate than other solutions, such as flowing horizontally above the microfluidic chip, under the same flow rate of cooling air. We investigated the key factors that influenced the heating and cooling rates for a specific microfluidic chip with three different heat resistor patterns. Through a comparison, it was found that Si (because of its good thermal properties and biocompatibility) was indeed suitable for materials such as microfluidic chips. At the same time, AlN and SiC have better thermal properties and can be considered microfluidic chip materials for experiments, but whether they are suitable for biological reactions needs further verification. They will benefit the future fabrication of related heat resistors and system integrations for PCR protocols.

## Figures and Tables

**Table 1:** The specific size of the PCR chip.

	Width (mm)	Length (mm)	Depth/Thickness (mm)	Diameter (mm)
Top (Glass)	21	56	0.5	/
Bottom	21	56	0.52	/
Resistor	/	/	0.01	/
Inlet/Outlet	/	/	0.75	2
Channel	0.6	4.32	0.25	/
Microcavity	/	/	0.25	3.5

**Table 2:** The specific parameters of the materials.

Material	Thermal Conductivity/(W/m·K)	Density/(kg/m^3^)	C_p_/(J/Kg·K)	Surface Emissivity
W	174	19,350	132	0.35 [22]
Cu	400	8940	385	0.1 [22]
Pyrex	1.38	2203	703	0.9 [22]
Si	130	2329	700	0.65 [23]
SiC	61,100/(T-115) (100~2300) [24]	3210	690	0.83~0.96 (149~649 °C) [22]
AlN	285	3230	600	Function related to temperature [25]
Al_2_O_3_	32	3980	850	0.42~0.26 (500~827 °C) [22]
SiO_2_	1.38	2203	703	0.85/0.42~0.33 (1010~1566 °C) [22]

**Table 3:** The key simulation results of 3 heaters.

	The Heating Rate (Si) °C/s	The Maximum Temperature Difference (Si) °C	The Heating Rate-Thickness of 5 μm (Si) °C/s	The Heating Rate-Thickness of 30 μm (Si) °C/s	With/Without Insulating Trenches (Si) °C/s
Heater 1	14.95	11.32	/	/	/
Heater 2	15.79	12.61	/	/	/
Heater 3	18.77	4.58	7.23	42.65	25.2/18.77

**Table 4:** The key simulation results of 3 cooling methods.

	The Cooling Rate with Insulating Trenches (Si) °C/s	The Cooling Rate without Insulating Trenches (Si) °C/s
Air Cooling 1	51.8	98.76
Air Cooling 2	48.44	99.24

## Data Availability

All data are presented in the main text of this manuscript.
